# Developing methods for the overarching synthesis of quantitative and qualitative evidence: The interweave synthesis approach

**DOI:** 10.1002/jrsm.1383

**Published:** 2019-12-13

**Authors:** Jo Thompson Coon, Ruth Gwernan‐Jones, Ruth Garside, Michael Nunns, Liz Shaw, G.J. Melendez‐Torres, Darren Moore

**Affiliations:** ^1^ College of Health and Medicine University of Exeter United Kingdom of Great Britain and Northern Ireland; ^2^ European Centre for Environment and Human Health University of Exeter United Kingdom; ^3^ Graduate School of Education University of Exeter United Kingdom of Great Britain and Northern Ireland

**Keywords:** evidence synthesis, methods, mixed methods, qualitative, quantitative

## Abstract

The incorporation of evidence derived from multiple research designs into one single synthesis can enhance the utility of systematic reviews making them more worthwhile, useful, and insightful. Methodological guidance for mixed‐methods synthesis continues to emerge and evolve but broadly involves a sequential, parallel, or convergent approach according to the degree of independence between individual syntheses before they are combined.

We present two case studies in which we used novel and innovative methods to draw together the findings from individual but related quantitative and qualitative syntheses to aid interpretation of the overall evidence base. Our approach moved beyond making a choice between parallel, sequential, or convergent methods to interweave the findings of individual reviews and offers three key innovations to mixed‐methods synthesis methods:

- The use of intersubjective questions to understand the findings of the individual reviews through different lenses,
- Immersion of key reviewers in the entirety of the evidence base, and
- Commencing the process during the final stages of the synthesis of individual reviews, at a point where reviewers are developing an understanding of initial findings.

Underlying our approach is the process of exploration and identification of links between and across review findings, an approach that is fundamental to all evidence syntheses but usually occurs at the level of the study. Adapting existing methods for exploring and identifying patterns and links between and across studies to interweave the findings between and across reviews may prove valuable.

**Highlights**

**What is already known?**

- Incorporating all dimensions of relevant evidence by combining qualitative and quantitative data in a single synthesis can provide greater insight than separate stand‐alone syntheses. Where applicable, incorporation of an economic perspective may be useful.

**What is new?**

- The interweave synthesis approach offers three key innovations:
  ○ The use of intersubjective questions to understand the findings of the individual reviews through different lenses,
  ○ Immersion of key reviewers in the entirety of the evidence base, and
  ○ Starting the process during the final stages of the synthesis of individual reviews, at a point where reviewers are developing an understanding of initial findings.

**Potential impact for Review Synthesis Methods readers outside the authors' field?**

- Adapting the fundamental methods used to explore and identify patterns and links *between and across studies* may be an effective approach to eliciting findings *between and across reviews*.
- Iteration of the methods for the overarching syntheses may be necessary as differences in the scope, range, and nature of the target body of available evidence for the separate reviews become apparent.
- Developing mechanisms within the team for the efficient sharing of understanding across the entirety of the evidence base may be particularly beneficial to the process.

## 1 INTRODUCTION

As a result of the Lancet REWARD campaign,^1^ many research funders now demand a systematic review of existing evidence before supporting further work.^2^ The incorporation of evidence from all relevant dimensions, not only “Does it work?” but “How or why does it work?” “What is the pre‐intervention context?” “Do people like it?” “Is it worth it?” and “How can it be implemented?” into one single synthesis can enhance the utility of systematic reviews making them more worthwhile, useful, and insightful. Alongside this, there is increasing recognition of the importance of qualitative evidence as a necessary component in understanding the impact and implementation of complex interventions and of health‐related decision making.^3^, ^4^ As systematic review methods are increasingly used to address questions other than those relating to the effectiveness of interventions, summarising what is already known is becoming more challenging.

Methods for mixed‐methods synthesis^5^, ^6^, ^7^, ^8^, ^9^ continue to emerge and evolve but broadly involve a sequential, parallel, or convergent approach^10^ according to the degree of independence between syntheses before they are combined. Approaches to synthesis may be inductive (theory building) where themes are derived from the data, or deductive (theory‐driven) where data are assigned to predefined themes. Several approaches, with accompanying exemplar reviews, have been described.^11^, ^12^, ^13^, ^14^, ^15^, ^16^, ^17^, ^18^, ^19^

A research and development agenda for systematic reviews that ask complex questions about complex interventions published by Noyes and colleagues in 2013 called for the publication of more exemplar reviews using novel approaches to mixed methods synthesis together with case studies of reviewers' experiences.^20^ Petticrew and colleagues highlighted the importance of evaluating and reporting the impact of changes to existing review approaches.^7^ This issue is further highlighted in a scoping review published in 2016^21^ which identified seven methods for integrating qualitative and quantitative evidence and concluded that further detail was required to allow for replication of the methods. This point was again reiterated by Harden and colleagues in the Cochrane Qualitative and Implementation Guidance series^5^ published in 2018.

Existing published examples rely on similarities between the research questions posed of the qualitative and quantitative evidence. In two recent projects, we needed to conduct mixed methods syntheses where the qualitative and quantitative questions did not directly map onto each other and/or where the inclusion criteria for the separate reviews were not closely matched. The aims of this paper are to reflect on two case studies in which we have used novel methods to maximise the value of existing evidence by combining the findings from quantitative and qualitative syntheses within an overarching synthesis. We aim to (a) explore some of the challenges experienced in applying existing mixed‐methods synthesis methods, (b) describe the methods we used, and (c) consider the strengths and weaknesses of the methods used by illustrating the extent of the additional insight we were able to provide, over and above what was possible with the separate reviews. In both cases, the aim of the overarching synthesis was to draw together the findings from the individual reviews to aid interpretation of the overall evidence base. In exploring our options for conducting the overarching syntheses and tailoring existing methods, we were cognisant of the complexities in the synthesis of different types of evidence.

## 2 CHALLENGES ENCOUNTERED IN APPLYING EXISTING METHODS FOR COMBINING THE FINDINGS FROM INDIVIDUAL REVIEWS

Quantitative and qualitative research has different aims, questions, and methods, and, therefore, different markers of study quality and potential sources of bias. For example, the aim of meta‐analysis is to test theory, and interpretation occurs largely before and after synthesis whereas the aim of meta‐ethnography is to generate theory, and interpretation occurs during synthesis to develop meaning.^22^ Therefore, although the reliability of the findings from quantitative reviews may be strengthened through greater frequency of occurrence, qualitative findings are strengthened by their ability to inform theory development and represent the complexity and depth of participant perceptions and meanings. Confidence in the findings of a qualitative synthesis involves the assessment of coherence, adequacy of data, and relevance in addition to consideration of methodological limitations.^23^

The strengths and weaknesses in study quality identified within each individual review, gaps in the evidence base and assumptions about the relationships, and shared meaning between unconnected studies are all factors we considered when attempting to synthesise across reviews. Given these limitations, we knew at the outset that the aim of the overarching syntheses would be to configure the evidence rather than to generate hypotheses, ie,that the overarching syntheses would only permit exploration of potential relationships between, and explanations for, review findings, and that any conclusions would remain tentative.

Many of the previously published examples of syntheses that combine separate quantitative and qualitative systematic reviews^16^, ^24^, ^25^, ^26^, ^27^ use the qualitative synthesis to inform, explain, enhance, extend, and/or supplement issues of interest in the quantitative review of effectiveness. This approach is reflected in the recently updated guidance from the Cochrane Collaboration Qualitative and Implementation Methods Group.^5^ In the most well‐known example of this, Thomas and colleagues^24^ used the findings from their thematic synthesis as a framework to juxtapose barriers, facilitators, and implied recommendations against the intervention evaluations reviewed quantitatively. The extent to which the interventions matched the implied qualitative recommendations was analysed alongside an analysis of whether or not interventions meeting such recommendations proved to be more effective or provided explanations of heterogeneity.

In another example where quantitative and qualitative research questions were different,^27^ a qualitative evidence synthesis was undertaken in order to explain a lack of evidence for or against the effectiveness of two approaches to tuberculosis intervention. The qualitative research questions focussed on the meanings that people attached to their experiences of tuberculosis and its treatment, and how these shaped their treatment uptake behaviour. Findings in the qualitative evidence synthesis were used to explain quantitative findings by describing potential barriers to uptake and differences between user group needs.

The similarity of the research questions posed across the evidence base is a fundamental difference between the individual reviews in these examples and the constituent reviews in our case studies. In both our case studies, there were important differences between the research questions (and hence the eligibility criteria for included studies) posed of the qualitative and quantitative evidence. Due to the differences in research questions and interventions that were included in each of the individual reviews, we felt that it would be limiting to use an approach where one review was used to explain the findings from the other review(s) only. Therefore, in both case studies, we used a combination of methods used in previously published mixed methods syntheses where they were relevant to our situation taking a dual line of inductive and deductive synthesis. In reflecting on our method, we also considered the conceptual schema proposed by O'Cathain and colleagues which describes three techniques for mixed methods synthesis—triangulation, following a thread, and the mixed methods matrix.^28^ Our approach moved beyond making a choice between parallel, sequential, or convergent methods to interweave the findings of individual reviews within the overarching synthesis. The methods we used draw from complexity theory, assuming that any intervention needs to be understood in terms of the wider environment and relationships within it^29^ and assumptions from mixed‐methods research that the process of engaging with divergent results from different viewpoints can yield a more comprehensive and nuanced understanding.^30^ At the crux of the methods described in our case studies is the use of intersubjective questions, rather than intersubjective answers. What we mean by this is that instead of using the answers to syntheses to seek areas of nuance and agreement between reviews, we framed the questions that would guide the synthesis in an intersubjective fashion, speaking to and across multiple bodies of evidence, and carried these through each review before reintegrating findings. Collaborative question‐and‐answer sessions, undertaken in both case studies, were essential to the formulation of intersubjective questions. Underpinning the development of intersubjective questions were two additional aspects of our approach: the immersion of reviewers in all aspects of the evidence base, as opposed to “siloing” quantitative and qualitative reviewers; and the commencement of integration as initial findings from each synthesis were being formulated, rather than waiting until one or more of the syntheses had been completed. Collectively, we regarded that these three aspects of our approach yielded an “interwoven” synthesis.

## 3 DESCRIPTION OF CASE STUDIES

Case study 1—*Nonpharmacological interventions for attention‐deficit/hyperactivity disorder (ADHD) delivered in school settings* consists of a series of four linked systematic reviews (two of quantitative evidence and two of qualitative evidence) to address the research questions shown in *Table* 1. The project was funded by the NIHR Health Technology Assessment Programme (10/140/02). The core review team comprised three experienced systematic reviewers (one with expertise in quantitative evidence synthesis, one with expertise in qualitative evidence synthesis and a third with experience in both quantitative and qualitative synthesis), supported by individuals with additional methodological expertise in quantitative and qualitative synthesis. At the point of undertaking the overarching synthesis, the team had been immersed in the data for approximately 12 months.

**Table 1:** Nonpharmacological interventions for ADHD in school settings—description of constituent reviews

Review description	Research questions	Type of included evidence	Synthesis method
Review 1: Effectiveness and cost‐effectiveness of interventions	Are nonpharmacological interventions delivered in school settings for children with or at risk of ADHD effective in improving (a) core ADHD symptoms (eg, inattention, hyperactivity), (b) ADHD‐related symptoms (eg, social skills), and (c) scholastic behaviours and outcomes (eg, achievement)? Is the effectiveness of these interventions moderated by particular programme features? Have these interventions been shown to be cost‐effective?	Quantitative evidence from 54 randomised clinical trials; no evidence to inform the assessment of cost‐effectiveness was identified	Random effects meta‐analysis and descriptive synthesis
Review 2: Attitudes towards interventions	What attitudes do educators, children with or at risk of ADHD, their peers, and their parents hold towards nonpharmacological interventions for ADHD used in school settings? Which school‐based nonpharmacological interventions for ADHD are preferred and how do attitudes towards these interventions compare to nonschool interventions including pharmacological ones? What factors affect attitudes held towards these nonpharmacological interventions (including children's ADHD subtype and teacher experience)?	Evidence from 28 studies that used quantitative data collection methods, eg, questionnaire and survey studies	Descriptive synthesis
Review 3: Experiences of interventions	What are the experiences of and attitudes towards ADHD interventions in school settings?	Evidence from 33 studies that used qualitative data collection methods	Meta‐ethnography
Review 4: Experiences of ADHD	What are the school‐related experiences and perceptions of pupils diagnosed with or at risk of ADHD, their teachers, parents, and peers?	Evidence from 34 studies that used qualitative data collection methods	Meta‐ethnography

Case study 2—*Improving the mental health of children and young people with long‐term conditions* consists of two linked systematic reviews (one of quantitative evidence and one of qualitative evidence) to address the research questions shown in *Table* 2. The project was funded by the NIHR Health Technology Assessment Programme (14/157/06). The core review team comprised two relatively inexperienced systematic reviewers with expertise in quantitative and qualitative research methods and a third with extensive experience of quantitative, qualitative, and mixed methods synthesis. Additional methodological expertise was provided by individuals with extensive expertise in quantitative, qualitative, and mixed methods synthesis. At the point of undertaking the overarching synthesis, the lead reviewer for each review had been immersed in the data for approximately 12 months and had a working knowledge of the findings emerging from the other review.

**Table 2:** Improving the mental health of children and young people with long term conditions—description of constituent reviews

Review description	Research questions	Type of included evidence	Synthesis method
Review 1: Effectiveness and cost‐effectiveness of interventions	1) What is the effectiveness and cost‐effectiveness of interventions aiming to improve mental health for children and young people (CYP) with long‐term conditions (LTCs) and symptoms of mental ill health? 2) What are the effects of such interventions on other key aspects of individual and family functioning?	Quantitative evidence from 25 randomised clinical trials; no evidence to inform the assessment of cost‐effectiveness was identified	Meta‐analysis and descriptive synthesis
Review 2: Experiences of interventions	1) What are the perceived effects of interventions aiming to improve mental health and wellbeing for children and young people (CYP) with long‐term physical conditions (LTCs) on mental health and other key aspects of individual and family functioning? 2) What are the factors that may enhance, or hinder, the effectiveness of interventions and/or the successful implementation of interventions intended to improve mental health and wellbeing for CYP with LTCs?	Evidence from 57 studies that used qualitative data collection methods	Meta‐ethnography

Full details of the methods used in the individual reviews are available elsewhere.^31^, ^32^, ^33^, ^34^

## 4 DESCRIPTION OF THE METHODS USED TO PRODUCE OVERARCHING SYNTHESES

### 4.1 Case study 1: Nonpharmacological interventions for attention‐deficit/hyperactivity disorder (ADHD) delivered in school settings

We worked inductively from the qualitative review findings about the experience of ADHD interventions and of ADHD in schools more generally to explore the complexity of the context in which nonpharmacological school‐based interventions for ADHD are used. We also worked deductively from the quantitative findings that describe the effectiveness of, and moderators for, interventions for ADHD in schools to consider potential relationships between moderators and effectiveness and examined how other findings might provide potential explanations and relevant information in response to them. In both cases, our aim was to identify qualitative findings that could provide potential explanations for the findings of review 1 (*Table* 1). We applied the approaches to the data iteratively and in parallel rather than sequentially allowing for the following limitations in the individual reviews: (a) in reviews 1 and 2, the poor methodological quality of some included studies was identified as a barrier to establishing effectiveness or comparing attitudes; (b) in review 3 analysis used by the majority of studies was mainly at a descriptive level; and (c) in review 4 important gaps in the literature were identified.

We undertook a five‐step process.

Step 1: Collaborative question and answer exercise

To enable a shared understanding of the links between the evidence in the reviews, we began the process with a collaborative question and answer exercise. An illustration of this process is provided in Box 1. Over a number of weeks, questions based on the findings of each review were generated and used to interrogate the other reviews for information that could potentially inform the findings or reveal gaps. The lead reviewer of each review developed questions, and the other reviewers responded to these questions (initially via written responses and then through discussion) from the perspectives of their reviews. Reviews 1, 3, and 4 were ready at the same time so we began by interrogating these; later we also explored similarities and differences between the findings of review 2 and the other reviews using a similar process. Questions were framed systematically using the format “Review 1 found X, can the other reviews inform these findings?” The resultant sets of questions and answers were appraised for plausibility and utility independently by the three reviewers in the context of what they knew from the reviewed material and later discussed.

**Box 1: Illustrations of the tables used in steps 1 to 3 of Case Study 1**

**Table:** 

Illustration of Step 1: the collaborative question and answer exercise		
Finding	Question	Answer
Review 2 considered an important tension acknowledged by teachers between the need to individualise interventions for children with ADHD whilst heeding their responsibility to other learners	Does review 3 recognise any issues that children with ADHD might experience due to individualised interventions, eg, stigma?	Only mentioned; stigma is usually linked with difference generally rather than interventions specifically, eg, due to constantly being in trouble, being different from peers

Illustration of Step 2: Identification of contextual elements that might influence the effectiveness of interventions		
Key categories	**Findings from review 3**: the attitudes and experiences of pupils, teachers, parents, and others using ADHD interventions in school settings	**Findings from review 4**: the experiences and perceptions of ADHD in school among pupils, their parents, and teachers more generally
Pupil‐level factors: identity, agency, process of stigma, and marginalisation		
Desire for approval	No relevant findings	Pupils wish to meet school expectations and are distressed and full of remorse that they cannot
Low self‐esteem/issues of identity	Low self‐esteem is seen as a problem for pupils with ADHD	ADHD is linked to negative impact on self‐esteem and developing identity
Agency	Pupils with ADHD held low self‐efficacy, attributing learning outcomes to circumstances beyond their control Studies noted the lack of agency seemingly experienced by pupils displaying ADHD symptoms during interventions and learning more generally	Many factors related to ADHD have the tendency to decrease pupil agency

Illustration of Step 3: Identification of hypotheses about the relationships between possible moderators and effectiveness of interventions Table a—a comparison of findings relating to effectiveness (or perceptions of effectiveness) from reviews 1, 3, and 4			
Scholastic behaviours and outcomes			
Outcome measure	**Findings from review 1**: the effectiveness and cost‐effectiveness of interventions	**Related findings from review 3**: the attitudes and experiences of pupils, teachers, parents, and others using ADHD interventions in school settings	**Related findings from review 4:** the experiences and perceptions of ADHD in school among pupils, their parents, and teachers more generally
Perceptions of school adjustment (teacher)	*d*_+_ = 0.26 (0.05 to 0.47)	There are negative attitudes to school and learning seen from pupils with ADHD	Negative attitudes to school
Curriculum achievement (child)	*d*_+_ = 0.50 (−0.06 to 1.05)	Some studies revealed that teachers and pupils with ADHD might be more interested in achievement than other outcomes	No relevant findings
d+, the difference between the means in each of two groups divided by their pooled SD (Cohen's d).			

Illustration of Step 3: Identification of hypotheses about the relationships between possible moderators and effectiveness of interventions Table b—potential moderators of intervention packages identified in review 1 with relevant findings from reviews 3 and 4				
Intervention package identified in Review 1	Definition of intervention package	Frequency of intervention packages and summary of corresponding moderator analyses from **Review 1**	**Review 3** relevant findings	**Review 4** relevant findings
Cognitive‐behavioural self‐regulation training	Establish methods for the child to self‐monitor and record their behaviour(s). Includes analysing the factors that lead to problem behaviour(s) and identifying solutions to overcome them (“problem solving”) and self‐instruction on how to perform the behaviour(s)	RCTs n = 10 Non‐RCTs n = 7 No evidence from moderator analysis that cognitive‐behavioural self‐regulation training has an impact on effectiveness	0/12 studies focussed on this intervention package; Teachers recognised difficulties of self‐regulation for pupils displaying ADHD symptoms	One study found pupils diagnosed with ADHD are often unaware of situations that precede or trigger loss of behavioural control; One study found pupils who become aware of such triggers are better able to take control of their learning

Illustration of Step 3: Identification of hypotheses about the relationships between possible moderators and effectiveness of interventions Table c—other potential moderators of the effectiveness of interventions in review 1, including delivery characteristics, participant characteristics, and study design with relevant findings from reviews 3 and 4.			
Source of heterogeneity	Findings from **Review 1**	Relevant findings from **Review 3**	Relevant findings from **Review 4**
Intervention delivery			
Setting within school: classroom vs all other settings	No evidence from moderator analysis that the setting for intervention delivery had an impact on effectiveness	Mixed teacher perceptions regarding benefit of withdrawing pupils from their classroom	No relevant findings

Step 2: Identification of contextual elements that might influence the effectiveness of interventions (inductive synthesis)

**Figure 1:**
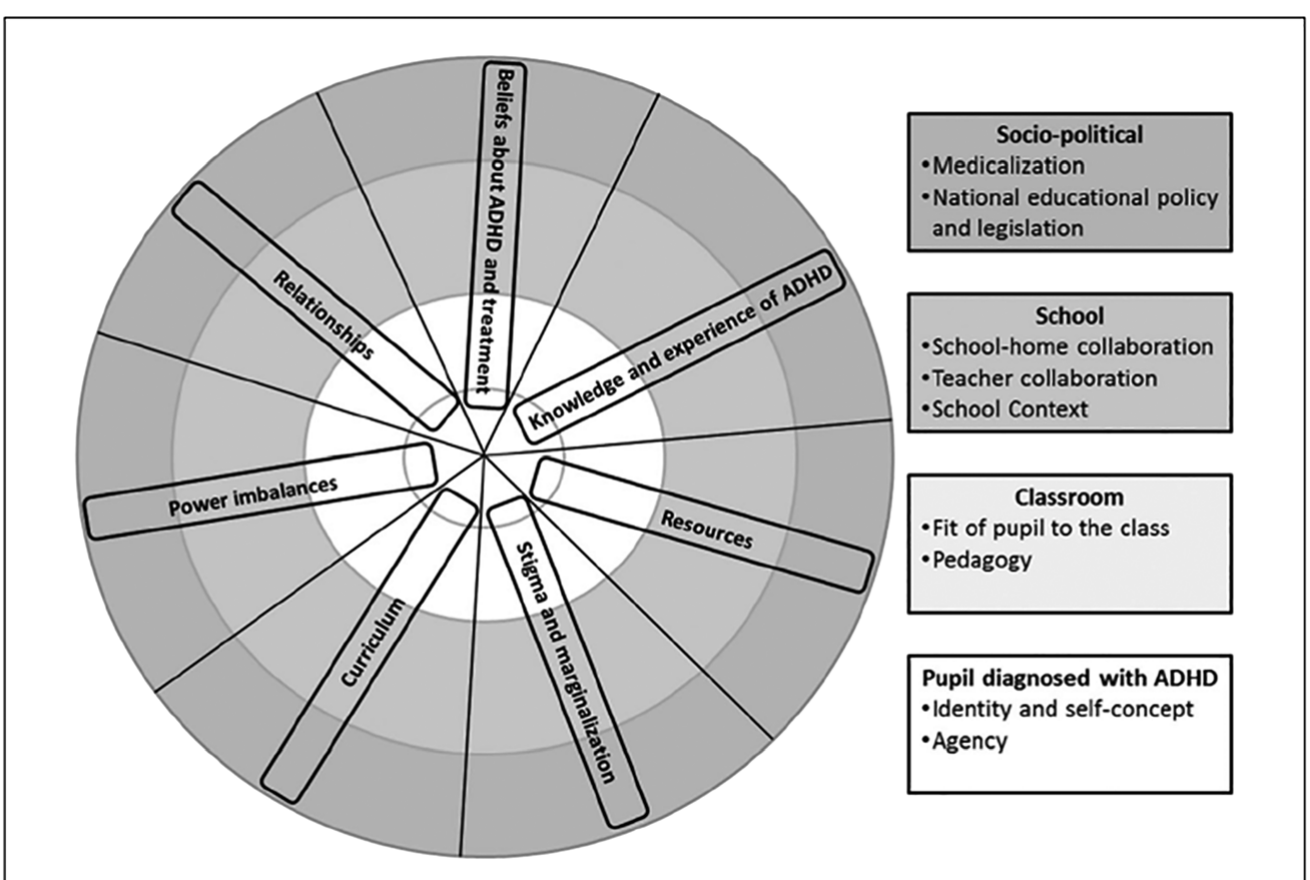
Conceptual model to represent a hierarchy of levels and key categories

Starting with the findings from reviews 3 and 4, the lead reviewers developed a coding framework derived from the question and answer exercise relating to the qualitative reviews. The reviews were coded in NVivo v.9.2 using this framework, and then short summaries of codes that appeared in each review were produced. These were reduced to short sentences and tabulated for the purpose of display. These code summaries were further analysed and refined, leading to the identification of four levels of context (pupil‐, classroom, school‐, and socio‐political‐level) and identification of key categories (eg, pupil knowledge about ADHD, teacher knowledge about ADHD, identity, agency, processes of stigma, and marginalisation) linking to each level across the qualitative reviews. This information was tabulated (as illustrated in Box 1) and a conceptual model (Figure 1) was created to represent a hierarchy of levels of context and key categories that might potentially influence the effectiveness of interventions for ADHD in schools. Finally, relationships between levels, key categories, and subthemes in the model and table were described.

Step 3: Identification of hypotheses about the relationships between possible moderators and effectiveness of interventions (deductive synthesis)

In this step, we started from the effectiveness findings and moderators for interventions reported in review 1 and considered whether the findings in reviews 2, 3, and 4 might offer hypotheses about the relationships between possible moderators and effectiveness. This was an iterative process requiring the creation of multiple additional tables to fully explore the potential relationships between the findings from the four reviews. Examples of additional tables created include: (a) a comparison of findings relating to effectiveness (or perceptions of effectiveness) from reviews 1, 3, and 4, (b) a table of the potential moderators of intervention packages identified in review 1 with relevant findings from reviews 3 and 4, and (c) a table of other potential moderators of the effectiveness of interventions in review 1, including delivery characteristics, participant characteristics, and study design with relevant findings from reviews 3 and 4. The tables formed the basis for in‐depth discussion between the lead reviewers of how the findings in reviews 2, 3, and 4 could inform, support, or contradict what was reported in review 1 and generate potential hypotheses about the relationships between possible moderators and effectiveness. Illustrative excerpts of the tables produced can be found in Box 1.

Step 4: Discussion of inductive and deductive syntheses

We then brought together the inductive and deductive syntheses in a structured narrative^31^, ^33^informed by the tables produced in steps 2 and 3 to highlight (a) the potential key relationships between possible moderators and the effectiveness of the interventions and (b) the complexity of the context in which the interventions are used.

Step 5: Validation of findings

Throughout the process we were triangulating the findings between reviews. We looked for situations where the potential hypotheses being generated did not fit the evidence from other perspectives. We also discussed the findings from the overarching review with multiple groups of different stakeholders^31^ to check the relationship of our narrative was familiar to their own experiences.

### 4.2 Strengths and weaknesses of this method

Our approach allowed comparison across four reviews seeking to address different but related research questions. The approach enabled us to draw together the findings from a wide breadth of evidence relevant to the use of ADHD interventions in the school setting. Through adopting both deductive and inductive methods and producing a conceptual model, we were able not only to use the qualitative evidence to help explain the effectiveness findings but were also able to consider potential relationships between moderators and effectiveness. Furthermore, focussing on the difference between the bodies of evidence emphasised issues that were discussed in the qualitative literature but were not considered or reported in the development or testing of interventions, providing opportunities for the review findings to contribute to the development and evaluation of future interventions.

The overarching synthesis enabled us to highlight a number of tentative implications that were not apparent from any of the separate reviews. These were categorised as (a) the context affecting interventions, (b) the development and evaluation of interventions, and (c) moderators of intervention effectiveness.^31^, ^33^

However, although we made attempts to construct valid comparisons across all reviews, our methods were not able to overcome some of the fundamental differences in the included studies resulting from differences in (a) research questions and (b) the nature of the evidence available to address the questions. These differences were most apparent in the inclusion of interventions across reviews which presented a challenge to integration. Considering such a wide variety of evidence in one overarching synthesis also highlighted disparities in the language used by researchers and educators. Whilst researchers reported the evaluation of discrete intervention packages in clinical trials, educators interviewed in qualitative studies also referred to ad hoc strategies used with pupils with ADHD as interventions. In drawing conclusions across the bodies of evidence, it is also important to acknowledge that in each separate synthesis and in the overarching synthesis, we made assumptions about relationships and shared meaning between unconnected studies.^35^

Practically, there were challenges in considering such a broad range of evidence in one overarching review. Although the lead reviewer for each review was well acquainted with the details of the evidence, and all reviewers had worked in part on all the reviews, it was not possible for all members of the review team to be completely immersed in all the evidence. Furthermore, as expected, some reviewers were more familiar with quantitative evidence and methods and others with qualitative evidence and methods. Efficient completion of the steps of the process within the time and resource limits was greatly facilitated by one member of the team (DM) being competent in synthesising both quantitative and qualitative evidence and functioning as a bridge between all four reviews. Full immersion of all the reviewers in the entirety of the evidence base would be extremely valuable but may not be realistic for many teams and projects.

### 4.3 Case study2: Improving the mental health of children and young people with long‐term conditions

We used solely deductive methods to draw together the findings from the two separate reviews (*Table* 2). We aimed to highlight areas where quantitative effectiveness data could help verify or refute suggestions put forward by the qualitative data; and where qualitative experience data could help to explain why an intervention may be effective or not.

**Figure 2:**
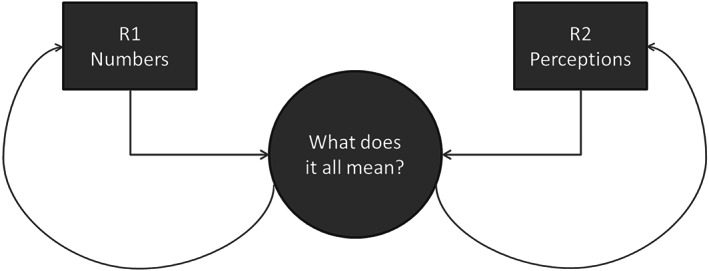
Case study 2 ‐ process of overarching synthesis

We undertook a four‐step process:

Step 1: **Collaborative question and answer exercise**—using a similar approach to the first case study, the core review team performed a collaborative question and answer exercise whilst the findings from the separate reviews were still preliminary. This allowed for the issues raised during the question and answer exercise to also contribute to the synthesis of the separate reviews (Figure 2). Questions were generated based on the findings of each review and the descriptive details of the included studies and used to interrogate the other review for information that could potentially inform the findings or explain gaps in the literature. The lead researcher for each review framed questions systematically using the format “Review 1 found X, can Review 2 inform these findings?”. Draft answers for each question were prepared and shared with the rest of the team. In answering the questions, reviewers consulted both the systematic review findings and the data extraction forms for included studies where necessary. Box 2 provides an illustrative excerpt of this process.

Step 2: Grouping of questions and answers into categories

The resultant sets of questions and answers were initially grouped into 13 categories according to shared ideas within the questions and/or answers. Through discussion and further consideration of the evidence, the sets of questions and answers were subsequently refined and condensed into nine categories. The final set of categories was: Degree of overlap between the reviews; Availability of up to date, good quality research; What works for whom?; Adaptations to interventions and flexibility; Accessibility and delivery of interventions; Stress and coping; Working with family and peers; Therapeutic relationships and Holistic approach.

**Box 2: Illustrations of the tables used in steps 1 and 3 of Case Study 2**

**Table:** 

Illustration of Step 1: the collaborative question and answer exercise	
Question derived from Review 2 (qualitative evidence) posed to Review 1 (quantitative evidence)	Response from Review 1 (quantitative evidence)
Is there any evidence for interventions tailored to the specific needs of the child being more effective than ones which are not?	Some evidence that programmes tailored to the LTC might be more effective than those which are not, eg, tailored to children of a certain age.
Question derived from Review 1 (quantitative evidence) posed to Review 2 (qualitative evidence)	Response from Review 2 (qualitative evidence)
Interventions in Review 1 were delivered either in a hospital/clinic, school, or at home/over the telephone. Does Review 2 suggest that the setting is a factor that affects the effectiveness of an intervention?	Yes, broadly the setting needs to be accessible and aspects of the setting may affect the extent to which an intervention is perceived to be engaging (see theme 1). Theme 2 regarding safe space implies that as well as the intervention staff, it is important that the setting is familiar and allows for a therapeutic atmosphere. A setting that allows for privacy and anonymity is seen as a positive thing in some studies and as such there are benefits of online interventions. The majority of Review 2 interventions were delivered in hospital/clinics (17 studies), very few were delivered at home, school or by phone. Online setting was next most frequent in 13 studies

Illustration of step 3: Description of categories and contribution of findings from each review				
Category	**Summary**	**Contribution from Review 1**	**Contribution from Review 2**	**Implications**
Accessibility and delivery of interventions	Considers the role of the setting, use of technology and flexibility of an intervention in ensuring that it can be accessed by children and young people with a long‐term condition	Some evidence that accessibility and familiarity if interventions may be beneficial, but difficult to tease out from other components of interventions	Familiar setting, use of technology and “therapists” who can relate to the needs of young people all perceived to be effective	Further research to investigate the impact of accessibility and delivery on the effectiveness of interventions is warranted

Step 3: Description of categories and contribution of findings from each review

Each category was written up in narrative form using the questions and answers as the initial basis for the content. This was an iterative process with additional ideas generated during the narrative process used to refine the categories and inform the synthesis of results within each individual review. Whilst lead reviewers for each review were fully conversant in the individual studies contributing to the findings from both reviews, the process was further supported by members of the wider review team less familiar with the evidence and able to challenge assumptions. Each category, relative contributions of each review to the categories, and the implications were discussed narratively and tabulated. An illustrative excerpt of this table can be found in Box 2.

Step 4: Validation of findings

Throughout the process, multiple reviewers and stakeholders discussed and compared ideas across studies and reviews to triangulate and explore findings between reviews. For this case study, the clinical stakeholders were particularly helpful in this regard, challenging and questioning our assumptions and interpretation.

### 4.4 Strengths and weaknesses of this approach

The process of bringing together findings from the two reviews required the immersion of both quantitative and qualitative researchers in the entirety of the evidence base. This approach not only allowed us to highlight clearer implications for practice and gaps for further research than was possible from the separate reviews but also strengthened the findings of the individual reviews by highlighting elements we had not previously considered.

As in Case study 1, integration was challenging due to the breadth of scope and the limited amount of overlap between the research questions and inclusion criteria (and therefore the evidence included) in the separate reviews. In the review of effectiveness, the population receiving the intervention were required to have elevated mental ill health at baseline; this inclusion criteria was not applied to the review of experiences due to the necessary differences in aims, objectives and methods of qualitative research. This resulted in marked differences in included evidence; the interventions in the review of experiences tended to focus on improving coping, stress, and self‐esteem, whilst the interventions in the effectiveness review aimed to improve symptoms of mental health disorders such as anxiety and depression. These differences precluded us from performing an inductive data‐driven synthesis. However, we were able to adapt the methods used in Case Study 1 and use deductive approaches to question the evidence from both the qualitative and the quantitative perspectives.

## 5 DISCUSSION

We have used two case studies to illustrate novel approaches to combine the findings from separate qualitative and quantitative evidence syntheses. In both examples, bringing together the findings from several reviews provided greater insight and understanding of the evidence than the separate reviews. We were also able to highlight clearer implications for practice and gaps for further research. The resulting outputs have more relevance and utility for decision makers who are unlikely to be interested in understanding whether something works without considerations of context, accessibility, and feasibility.

Our approach moved beyond making a choice between sequential, parallel, or convergent methods to interweave the findings of individual reviews within the overarching synthesis. This interwoven approach, which involved intense debate and discussion and relied heavily on teams working together across the evidence bases, offers three key innovations to mixed‐methods synthesis methods:

- The use of intersubjective questions to understand the findings of the individual reviews through different lenses,
- Immersion of key reviewers in the entirety of the evidence base, and
- Starting the process during the final stages of the synthesis of individual reviews, at a point where reviewers were developing an understanding of initial findings.

### 5.1 Implications

The benefits of combining the findings from quantitative and qualitative syntheses have been described and recognised by other authors,^5^, ^6^, ^7^, ^8^ and there is methodological guidance available.^5^, ^11^, ^12^, ^13^, ^14^, ^15^, ^16^, ^17^, ^18^, ^19^ Underlying our methods in both examples is the process of exploration and identification of links between and across review findings; an approach that is fundamental to all evidence syntheses but usually occurs at the level of the study. Adapting existing methods for exploring and identifying patterns and links between and across studies to interweave the findings between and across reviews may provide a valuable means of synthesis across reviews.

In practice, producing the overarching syntheses was challenging due to necessary differences in the focus of research questions and the scope, range, and nature of the target body of evidence available. Some of the challenges were apparent at the outset of the individual reviews, but others emerged as the reviews progressed. This has practical implications for the protocol stage of a review as iteration of the methods for the overarching synthesis as the separate reviews near completion precludes comprehensive methodological description in a protocol before the separate reviews are started. As in qualitative synthesis protocols, it may be preferable to determine synthesis methods having first identified the nature and content of the separate reviews.^36^

Enabling a full understanding of the breadth of evidence contributing to the overarching synthesis across as many members of the review team as possible greatly facilitated the process in both case studies. In our experience, reviewers tend to be familiar and comfortable with either the quantitative evidence or the qualitative evidence in an evidence synthesis—developing mechanisms within the team for the efficient sharing of understanding across the entirety of the evidence base was particularly beneficial to the process.

Improving the reporting of synthesis methods is essential if we are to advance methodological innovation in this area. The lack of transparency of reporting of synthesis methods is well recognised^21^ and can be especially evident where narrative synthesis methods have been used due to the potential for author interpretation.^37^ A protocol for a mixed methods study to develop a reporting guideline for narrative synthesis was published in February 2018.^38^

### 5.2 Strengths and weaknesses

This paper illustrates three methodological innovations in mixed‐methods synthesis which have been used, adapted and developed in two different contexts. We continue to reflect on and develop an interwoven approach to overarching synthesis as we apply the approach to other syntheses. Both case studies involved three core team members (D.M., R.G., J.T.C.) who have worked together for a number of years and have a similar position on evidence synthesis, a shared respect for the equal but different value of quantitative and qualitative evidence in understanding complex scenarios and an understanding of our collective responsibility to make the most of available evidence.

## 6 CONCLUSION

Incorporating all dimensions of relevant evidence by combining qualitative and quantitative data in a single synthesis can provide greater insight than separate stand‐alone syntheses. An interwoven approach to mixed‐methods synthesis which necessitates understanding of the evidence through different review lenses may provide a valuable means of synthesis across and between reviews. Greater transparency in reporting the rationale and methods for synthesis is necessary to advance methodological innovation.

## COMPETING INTERESTS

None to declare.

## FUNDING SOURCE

Preparation of this manuscript was supported by the National Institute for Health Research (NIHR) Collaboration for Leadership in Applied Health Research and Care South West Peninsula. The projects in which the methods were developed were funded by the NIHR Health Technology Assessment Programme (Project number HTA 10/140/02 and HTA 14/157/06). The views expressed are those of the author(s) and not necessarily those of the NHS, the NIHR, or the Department of Health and Social Care.

## DATA SHARING

Data sharing is not applicable to this article as no new data were created or analysed in this manuscript.

## CONTRIBUTORSHIP

All authors participated in the conception and design of the article, worked on the drafting of the article and revised it critically for important intellectual content, and approved the final version to be published.
